# The Mechanisms of Developing Fungicide Resistance in *Fusarium graminearum* Causing Fusarium Head Blight and Fungicide Resistance Management

**DOI:** 10.3390/pathogens13111012

**Published:** 2024-11-18

**Authors:** Malini Anudya Jayawardana, Wannakuwattewaduge Gerard Dilantha Fernando

**Affiliations:** Department of Plant Science, University of Manitoba, Winnipeg, MB R3T 2N2, Canada; malini.jayawardana@umanitoba.ca

**Keywords:** Fusarium head blight, *Fusarium graminearum*, fungicide, fungicide resistance, mutation, over expression, efflux pumps

## Abstract

Fusarium head blight (FHB), primarily caused by *Fusarium graminearum*, is one of the economically significant diseases in small grains. FHB causes severe damage to wheat production and grain quality. Several management strategies have been developed to control FHB, and chemical control through fungicides plays a significant role. Although fungicides have effectively controlled *F. graminearum* in the field, the continuous exposure causes a selection pressure in the pathogen population towards fungicide resistance. Several studies have identified fungicide-resistant *F. graminearum* isolates and fungicide-resistance mechanisms. Although new fungicides with a new mode of action can be introduced into the market, developing a new fungicide is time-consuming, and extra efforts are needed for testing, approvals, and registrations. Therefore, it is essential to strategize the methods to delay the fungicide resistance. This review focuses on the impact of several fungicide applications currently used on FHB, focusing on *Fusarium graminearum*, the status of the fungicide sensitivity for fungicide classes, the resistance mechanisms against fungicides, and the mitigation strategies to delay the development of fungicide resistance in the pathogen population. Studying the fungicide resistance mechanisms and the mitigation strategies will be helpful in the future to use the available fungicides against *F. graminearum* without losing its effectiveness.

## 1. Introduction

Fusarium head blight (FHB) is one of the most devastating diseases that causes significant yield losses in small grains, including wheat, barley, oats, and corn worldwide. Although several other Fusarium species are associated with FHB, *Fusarium graminearum* is considered the major pathogen causing FHB [1,2,3,4]. The symptoms of FHB can be characterized as blighted and shrunken wheat heads resulting in light-weight kernels [3]. In addition, the fungal sporodochia and perithecia can be visualized as pink and purple on glumes and seeds. The pathogen-infected seeds are called tombstones and Fusarium-damaged kernels (FDKs) [3].

The most devastating effect of FHB is the accumulation of mycotoxins, such as trichothecenes, including deoxynivalenol (DON) and its derivatives (3-acetyl deoxynivalenol (3-ADON), 15-acetyl deoxynivalenol (15-ADON)), NX-2, NX-3, and nivalenol (NIV) [5,6,7]. In addition, the mycotoxin zearalenone (ZEA) is also produced by several species of Fusarium including *F. graminearum* [6,8,9,10]. Altogether, these mycotoxins can directly impact both humans and animals [11]. The toxin accumulation results in downgrading, directly impacting marketing, processing, and exporting [1]. Most importantly, these mycotoxins can induce protracted effects in humans and animals, including vomiting, feed refusal, bleeding, and dizziness [12,13]. These toxins also impact plants, resulting in wilting, necrosis, and chlorosis [14]. With these implications, FHB is considered an economically significant disease in many wheat and small grain growing areas worldwide, including North America.

Since FHB is an economically significant disease, effective management techniques should be practiced to control FHB in fields. Host resistance, chemical control using fungicides, and cultural practices such as crop rotation are essential in controlling FHB in the field. Among them, host resistance is important but mostly has been found in bread wheat, and less resistance has not been found in durum wheat yet [15,16,17]. However, the major resistance genes found in bread wheat, such as *Fhb1* and *Fhb7,* were successfully integrated into durum wheat to improve the host resistance in the durum background [15,18]. Besides the resistance genes identified so far, several genes associated with morphological traits such as *Rht,* carrying a semi-dwarfing allele, vernalization requirement genes, *Vrn,* and another extrusion gene, *Qfhs.ifa-5A,* were also reported to influence FHB resistance [19,20,21].

Chemical control through fungicides has been identified as an effective strategy for controlling FHB. Several fungicide classes, such as demethylase inhibitors (DMIs) and Quinone outside inhibitors (QoIs), have been registered as effective fungicides for managing FHB [22,23]. Although fungicides have effectively controlled FHB in the field, the overuse of fungicides brings many drawbacks to the growers and wheat industry. One example is the fungicide-resistant pathogen population that results from continuous exposure to fungicides over the years. This review addresses the causes of fungicide resistance in the pathogen population and the mitigation of fungicide resistance in the pathogen population.

## 2. FHB Control by Fungicides

Chemical control is one of the effective ways to control FHB. It has been reported that the fungicides can reduce the FHB level by 77% and the mycotoxin level by 89% [24]. Fungicides can be used as one of the common alternatives where host resistance is lacking, and they have been used together with host resistance. The concept of chemical control to control diseases was introduced in the mid-1800s with the introduction of Bordeaux mixture and lime sulfur [25]. Since then, fungicides have been developed by targeting specific sites and multiple fungal growth and metabolism targets. Presently, many commercial fungicides targeting different modes of action are available on the market. However, the International Fungicide Resistance Action Committee (FRAC) has grouped the fungicides by focusing on the mode of action and resistance risk [26]. According to FRAC, there are several modes of action based on nucleic acid metabolism, cytoskeleton, and motor protein, respiration, amino acid, and protein synthesis, signal transduction, lipid synthesis, cell wall biosynthesis, cell wall melanin synthesis, host plant defense induction, unknown mode of action, chemicals with multisite activity, and biologicals with multiple modes of actions [26]. Table 1 shows several fungicides used for *F. graminearum*, their mode of action, and the FRAC codes. In addition, FRAC has assigned a specific code for each group, and this is helpful for the growers to decide the fungicides they want each year to assist and prevent fungicide resistance in their fields [25].

Although many commercial fungicides are available in the market, they should be registered for each disease, and the fungicides assigned for FHB control vary from country to country. For instance, DMIs are registered in Canada to control FHB in the field. Currently, four common DMI fungicides, including ‘Prosaro-active ingredients; prothioconazole + tebuconazole’, ‘Caramba-active ingredient; metconazole’, ‘Folicur-active ingredient; tebuconazole’, and ‘Proline-active ingredient; prothioconazole’ have been used commonly in Western Canadian fields [27]. In China, DMIs and QoIs are common fungicides used for mitigating FHB, and in Italy, DMIs and QoIs have been registered to mitigate FHB [24]. Fungicide applications have been used in most wheat-growing areas worldwide, including China, the United States, Russia, India, and Canada. Many studies support the positive impact of fungicide use on wheat yield and grain quality by reducing the FHB pressure [22,24]. For instance, the study conducted in Italy investigated the effect of common fungicides, including DMIs and QoIs, against the development of FHB, DON accumulation, and yield [24]. The results support that the yield and the thousand-grain weight are higher in all the fungicide-applied field plots than in the controls. In addition, the DON content in the fungicide-treated wheat samples was significantly lower than in the fungicide-non-treated plots. Thus, this proves that these fungicides commonly used in Italy can considerably reduce the FHB and mycotoxin accumulation, allowing for a high yield. Likewise, several other European studies found that many fungicides such as DMIs and SHDIs control the FHB pressure with different efficacies [43,44]. This was the same in other wheat-growing regions in the world, including China, Canada, the United States, and India, where fungicides significantly reduce FHB impact by reducing mycotoxin levels and increasing yield [27,28,29,45,46,47]. It has been reported that tebuconazole, a DMI fungicide, can lessen the FHB severity by 25–77% and reduce DON accumulation by 32–89%. The fungicide prothioconazole also significantly controls FHB, reducing the FHB severity by 39–93% and decreasing the DON accumulation by 40–90% [48,49,50]. Although many studies found that fungicides positively impact FHB control, a few studies provide contradictory results where the use of fungicides reduces FHB levels but increases or has no impact on DON levels [51,52,53,54]. For instance, it has been reported that DMI fungicides such as tebuconazole effectively controlled the pathogen and reduced the DON level in the samples. In contrast, some QoI fungicides, such as azoxystrobin, did not significantly impact controlling the pathogen but increased DON production [24,51]. However, no evidence has been found yet that fungicides directly stimulate DON production [51,52]. More studies should be conducted to confirm this.

Although fungicides can control FHB levels effectively in the field, several factors are important to consider in obtaining better results from fungicide use. For example, fungicide application time is crucial to obtain better results. Since the pathogen can infect during the anthesis, applying the fungicides in early anthesis is recommended, and the window can be extended up to a few days post-anthesis [55,56]. However, several studies have identified that the timing of fungicide application largely depends more on the time of *F. graminearum* infection than on wheat phenology [56,57]. In addition to fungicide application timing, it is essential to apply fungicides with minimal frequency, which is enough for FHB control in the field. This will be further discussed under Section 5 in this review. These protocols have been followed to keep the fungicide application level minimal but to control FHB effectively. Otherwise, the overuse of fungicides in the field harms the wheat growers and industry, which brings fungicide resistance.

## 3. The Development of Fungicide Resistance in Pathogens

Although fungicides can effectively control FHB, overuse substantially negatively impacts FHB control. One is developing fungicide resistance mechanisms in the pathogen population. Fungicide resistance can be defined as the acquired and inheritable reduction developed in the fungus against a specific anti-fungal agent/agent [Background information, www.frac.info [58]]. When the fungicides are applied in a controlled manner, the possibility and frequency of evolving resistant pathogens in the open field are relatively low and have minimal/no negative impact on disease control. But once the same fungicides are used over the years extensively, then it creates selection pressure among the pathogen population, and this leads to predominate the fungicide-resistant individuals over sensitive individuals in the pathogen population, and ultimately, that fungicide ends up as ineffective in controlling the pathogen population in the field [59]. In addition to the overuse of fungicides, other factors, such as the fungicide’s mode of action, epidemiology, the biology of the pathogen, and other agricultural practices in the field, also affect the building up of a fungicide-resistant pathogen population [59]. Although the selection pressure builds up the fungicide-resistant population, several studies have been reported about the fitness defects of the pathogens associated with fungicide resistance. For instance, Wen et al. [40] found that the fludioxonil-resistant isolates carry fitness defects in mycelial growth, conidiation, and virulence. Another study by Wen et al. [41] found that the *F. graminearum* mutants with dual resistance to fludioxonil and phenamacril fungicides have shown fitness defects on mycelial growth, conidiation, DON production, and virulence. Although the selection pressure favors the existence of the resistant population in the field, the adaptation features of the fungi and the environmental heterogeneity lead to the coexistence of both fungicide-resistant and sensitive isolates for a prolonged time [60,61]. This is important for the management perspective where the fungicide-sensitive population’s existence, even in a small proportion, is important to enhance the recovery of the fungicide-sensitive population in the future and further helps to enhance the longevity of using fungicides [60].

The fungicide resistance that develops in pathogens can be grouped into several significant mechanisms. They are conferred as 1. alteration in the target site, 2. overexpression of the target protein, 3. having an alternative metabolic pathway to evade the process inhibited by the fungicide, 4. metabolic breakdown of the fungicide in the pathogen, and 5. exclusion or active transport of the fungicide [62,63,64].

**Figure 1:**
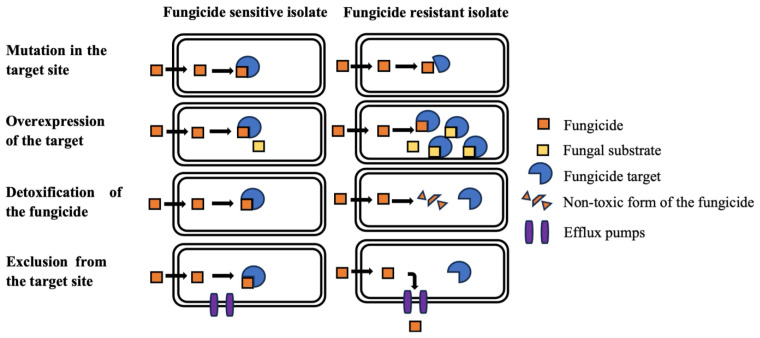
The fungicide resistance mechanisms developed in the pathogen. The diagram shows how the sensitive and fungicide-resistant isolates react in the presence of fungicides.

Among the fungicide resistance mechanisms, the most common way of having resistance in fungi is the alteration of the fungicide target site. This alteration is achieved in the fungal genome by generating mutations in the target site during DNA replication. These mutations change the amino acid sequences, thus resulting in the altered shape of the target site (Figure 1). Hence, the fungicide cannot fit with the target site, which reduces sensitivity to the fungicide [58,65]. The detoxification of the fungicide is primarily performed by modifying the metabolic machinery of fungi. Thus, it leads to the result of a nontoxic form of the fungicide in the fungal body, and it leads to reduced sensitivity to the fungicide (Figure 1) [58]. Overexpression of the target site is another way to bring resistance to the fungal body (Figure 1). In general, there is competition between the fungicide and natural substrate produced by fungi at the target site, and the failure of the natural substrates to compete with the fungicide results in sensitive isolates. However, in the presence of overexpressed target sites in the fungal body, the pathogen’s natural substrate has less competition to the target site, leading the fungus to bind its natural substrate to the target site enzyme (Figure 1). This leads to maintaining cellular respiration normally. Therefore, overexpression helps the pathogen’s survival to a certain extent, resulting in resistant isolates [58,66]. The final mechanism that fungi exerts is excluding the foreign substances by efflux pumps (Figure 1). Naturally, this efflux system protects the fungal body from foreign substances and toxic compounds. These materials are transported outside the fungal cell by transporters such as ABC and MFS transporters; thus, they protect the fungal growth and development by maintaining regular mechanisms. Generally, these efflux pumps fail to pump fungicide compounds out of the cells, resulting in sensitive isolates. However, the resistant fungal isolates can pump the fungicide compounds through fungicide transporters out of their cell [58,62]. In addition to these mechanisms, unidentified mechanisms may also be associated with fungicide resistance in pathogen populations [58]. In addition, one or more mechanisms can also be developed in a pathogen against fungicides.

## 4. Reports of Fungicide Resistance and Common Fungicide Resistance Mechanisms Found in *Fusarium graminearum* Species Complex

It has been reported that certain fungicide classes, including MBC fungicides, DMIs, and phenylpyrroles, are used to improve grain yield and FHB disease severity in the field [67,68]. However, the effect of QoIs in controlling FHB is still doubtful [69]. Although these fungicides efficiently control FHB in the field, some incidences have been reported on reducing the sensitivity of the pathogen against fungicides. In China, the use of benzimidazoles against FHB started in the 1960s, and they have used benzimidazoles during the period of wheat heading and flowering against FHB [33,70]. However, using these fungicides over many years has resulted in resistance to benzimidazoles in the pathogen population. For example, the study by Liu et al. [40] reported that out of 1132 isolates in China collected from a three-year field survey, it includes 31 resistant isolates to carbendazim and other benzimidazoles. In China, several other studies have also reported on the benzimidazole resistance in FHB causative agents collected from different wheat-growing provinces in China [33,35,36]. The most common resistance mechanism found in *F. graminearum* against benzimidazoles is the point mutation in the β_2_-tubulin gene at different codons 167, 198, and 200 [70,71,72,73]. However, the study conducted by Chen et al. [33] found benzimidazole-resistant *F. graminearum* isolates, but no mutations were present on the target gene β tubulin. This indicates that other resistance mechanisms also exist in *F. graminiearum* against benzimidazoles. According to Qui et al. [74], an overexpression of β_2_-tubulin was reported as explained by the increment of mRNA level with the benzimidazole resistance in *F. graminearum*.

DMIs are another common fungicide that majorly controls FHB in many wheat-growing areas, including China, the United States, Brazil, and Canada [27,28,30,31,32]. In China, DMIs have been used widely in wheat fields as an alternative to benzimidazoles, and benzimidazoles have been identified as ineffective fungicides in controlling FHB lately with the increasing number of benzimidazole-resistant pathogen isolates [28]. Although DMIs effectively control FHB in many wheat-growing areas, there are some incidences where the pathogen population has insensitivity to DMI fungicides in certain areas, including the United States and China [31,75,76]. According to Spolti et al. [31], the first field *F. graminearum* isolate was found to be resistant to one of the DMI fungicides, tebuconazole, in the United States. Likewise, several studies have been reported about DMI insensitivity or resistance in several wheat-growing regions in the world, including Europe and Asia [77,78,79,80]. To date, DMIs are performing better than benzimidazoles; therefore, fewer reports are available on DMI-resistant or insensitive isolates naturally occurring in wheat-growing fields [81]. However, studying the DMI-resistant mechanisms in the FHB pathogen population is still important. Since a few isolates are available as DMI insensitive or resistant in the field, many studies have been focused on creating DMI-resistant/insensitive isolates under laboratory conditions and using them to study the resistance mechanisms [77,78,81,82]. With the aid of laboratory-induced mutants for DMI resistance/insensitivity, several studies reported about potential resistance mechanisms established for DMI resistance. Amino acid substitutions which help to alter the target site and overexpression of the target are identified as common resistance mechanisms in *F. graminearum* for DMI resistance [78,81,83]. The study conducted by Zhou et al. [82] used laboratory-induced mutants for DMIs and found several point mutations present in the mutants associated with DMI resistance, and, among them, the amino acid substitutions of S28L, S256A, and V307A in the target homolog *CYP51C* were consistently present in DMI resistant mutants. The laboratory-induced ketoconazole-resistant isolates have several amino acid substitutions, including G443S, D243N, or combined mutations E103Q&V157 L on one of the homologs of the target gene *CYP51A* [81]. However, this same study found no mutations in the other two homologs, *CYP51B* and *CYP51C*. In addition, overexpression was also observed on the same mutants where the amino acid substitutions occur on *CYP51*, but the overexpression of the *CYP51* homologs was different based on the mutants. For instance, the overexpression of all three homologs was observed in the mutant having D243N substitution, and the combined mutant (E103Q and V157L) had the overexpression of two homologs (*CYP51A* and *CYP51B*). In contrast, the mutant containing G443S amino acid substitution has overexpression only in the *CYP51A* homolog [81]. Furthermore, the experiments performed on fitness penalty revealed that the mutant G443S has no fitness penalty. Although Duan et al. [81] found the same mutant has both mutation and overexpression resistance mechanisms to DMIs, the study conducted by Liu et al. [59] found no mutations in the *CYP51* gene in the DMI-resistant mutants, and only the overexpression of *CYP51A* and *CYP51B* was observed. Therefore, there might be one or more than one resistant mechanism associated with fungicide-resistant isolates. Therefore, testing all the possible resistant mechanisms when studying fungicide-resistant isolates is necessary. According to Yin et al. [78], the DMI-insensitive/resistant mutants did not show any point mutation or overexpression of the target site, suggesting that additional resistant mechanisms other than point mutations and overexpression exist with DMI-resistant isolates. To support this scenario, it has been reported that the ATP binding cassette (ABC) transporters are responsible for the DMI tolerance of *F. graminearum*, suggesting that the efflux pumps also play an important role in DMI resistance in *F. graminearum* [23,84]. In addition, the study conducted by Becher et al. [79] found that the multidrug-resistant *F. graminearum* isolates and mostly the multidrug resistance-related mechanisms are linked with the activation of the efflux pumps. Likewise, several studies have reported that the efflux pumps in *F. graminearum*, such as ABC transporters, significantly reduce sensitivity to DMI fungicides [85,86]. According to Ma et al. [86], a plasma membrane located H^+^ antiporter, FgQdr2, is responsible for being an efflux pump associated with multidrug resistance in *F. graminearum*. In addition, it has been reported that the other causative agents of FHB also have developed fungicide resistance mechanisms. For example, it has been reported that the laboratory-induced *F. culmorum* strains have shown some resistance to DMI fungicides, and the potential resistance mechanism was identified as the overexpression of the ABC transporters [87]. In addition, it has been found that resistance to fungicides occurs through the interaction of several pathways. For instance, the study conducted by Wang et al. [88] found that the sensitivity to a DMI fungicide, tebuconazole, in *F. graminearum* can be altered synergistically by regulating calcium–calcineurin and high osmolarity glycerol pathways. However, DMI resistance in the pathogen through detoxification has not yet been reported. In Canada, DMI has been extensively used to control FHB. Four common DMI products used in Canada are ‘Prosaro (active ingredients; prothioconazole + tebuconazole)’, ‘Caramba (active ingredient; metconazole)’, ‘Folicur (active ingredient; tebuconazole)’, and ‘Proline (active ingredient; prothioconazole)’. Each has a different combination of active ingredients [27]. Although DMI fungicide resistance is found to be rare in Canada, it is important to monitor the sensitivity of the pathogen population over the years. Monitoring the pathogen population for fungicide sensitivity is crucial to take precautions before fungicide resistance becomes a huge issue in Western Canadian wheat fields.

Like MBC fungicides, another standard fungicide class that was identified as ineffective against FHB are QoIs [22,89]. Unlike DMIs, natural resistance for QoIs in pathogen populations can be found. For example, the study conducted by [90] performed QoI fungicide sensitivity tests for *F. graminearum* isolates collected from different regions in the world, including Belgium, Canada, Germany, Italy, Luxembourg, and the United States, and all the isolates tested were insensitive to QoIs. Common point mutations have been found against QoI resistance, including F129L, G137R, and G143A in *Cytochrome b* [91,92]. However, these common point mutations were reported to be absent in QoI-resistant *F. graminearum* isolates so far and the resistance mechanisms for QoIs remain unclear for *F. graminearum* [22,93]. It has been reported that another species of *Fusarium*, *F. pseudograminearum*, the causative agent of Fusarium crown rot, has the amino acid substitution G143S, but no apparent point mutations were reported in *F. graminearum* QoI-resistant isolates [94]. Although clear resistant mechanisms have yet to be discovered in QoI-resistant *F. graminearum* isolates, other resistance mechanisms, such as the upregulation of efflux pumps, are reportedly involved with QoI resistance in *F. graminearum* isolates resistant to QoIs. For instance, the study by Thurau et al. [89] found four transporter genes, two belonging to the MFS transporter family and one to the ABC transporters and polypeptide transporters, were highly upregulated with QoI exposure. This suggests that the QoI resistance in *F. graminearum* is governed by the expression of efflux pumps, which transport fungicides out of the fungal cell body.

Since the benzimidazole-based fungicides are less effective in controlling FHB in China, several alternative fungicides have been introduced, and cyanoacrylate-based fungicides are among them. This was introduced into the market, and the efficacy of controlling FHB with this fungicide was better than the traditional benzimidazole fungicides [37]. However, it has been found that an actin-bundling protein in *F. graminearum* was found to be associated with the resistance to this new fungicide, JS399-19 [95]. In addition, several other studies have also reported on fungicide resistance mechanisms and responsible genes/factors against cyanoacrylate fungicides, such as phenamacryl [41,96,97,98]. The study conducted by Liu et al. [96] revealed that the transcription factor, FgTfmI, regulates the expression of the genes associated with phenamacryl tolerance in *F. graminearum* such as *FgMYO1.* Another study conducted by Zheng et al. [99] found certain point mutations at the codon 216, 217, 418, 420, or 786 at the phenamacryl target gene, *Myosin-5*. To validate whether these mutations are associated with phenamacryl resistance in *F. graminearum*, the myosin-5 loci were exchanged between the phenamacryl resistant and sensitive isolates, and it was found that the isolates having resistant fragments were resistant to phenamacryl [99]. The study conducted by Bao et al. [97] performed a computational approach to identify potential mutations in *F. graminenarum* associated with phenamacryl resistance and found that the mutation of C423A in the phenamacryl target gene *Myosin-1* was associated with phenamacryl resistance by impairing the binding of fungicide phenamacryl with its target [97]. In addition, laboratory-induced mutants for cyanoacrylate resistance were found to be resistant to both benzimidazoles and cyanoacrylates, which creates double resistance to both fungicides benzimidazoles and cyanoacrylate [34]. Therefore, this is a good example to think that it is important not only to focus on developing new fungicides, but to use them effectively to delay the development of resistance in the field.

It has been known that the fungicide succinate dehydrogenase inhibitors (SDHIs) are also registered for FHB control in several countries, including China [23,100]. However, field isolates showing SDHI resistance were also found to lead to the necessity of studying the resistance mechanisms associated with SDHI fungicide resistance [38,39,101,102,103]. Several studies have investigated the potential resistance mechanisms of SDHI resistance in *F. graminearum* [39]. The mechanism associated with the natural resistance against SDHI fungicides in *F. graminearum* was studied by Sun et al. [38], who found that a paralog of succinate dehydrogenase subunit C (*FgSdhC_1_*) is important to have natural resistance, where a single nucleotide variation leads to a premature termination codon, resulting in the failure of the function by *FgSdhC_1_*, which leads to natural resistance in *F. gramineaum*. The resistance for SDHI fungicides was also studied with the aid of laboratory-induced mutants [39,102]. The study conducted by Miao et al. [39] identified several potential point mutations conferring resistance to pydiflumetofen, an SDHI fungicide, and the potential mutations are H248Y and A73V located in *FgSdhB* and *FgSdhC_1_* genes, respectively. Another study conducted by Zhou et al. [102] investigated the resistance mechanisms for the same fungicide used in Miao et al. [39] and found several point mutations of Y182F in the subunit *FgSdhA*, H53Q, C90S, and A94V in subunits *FgSdhB* and S31F in *FgSdhC,* commonly found on laboratory-induced SDHI mutants. Although several mutations were observed against SDHI fungicide in *F. graminearum*, no cross-resistance was found in pydiflumetofen with other fungicides tested, including azoles, phenylpyrrole, QoIs, and benzimidazoles. In another study, Sun et al. [103] found several laboratory-induced mutants for SDHI resistance and found several point mutations associated with resistance but, interestingly, the mutant contains A83V in the FgSdhC subunit, reducing the efficacy of the fungicide pydiflumetofen by 42.7%, concluding that there is a potential to have a moderate risk of developing resistance in *F. graminearum* for the SDHI fungicide pydiflumetofen. Altogether, it is important to study the potential fungicide resistance mechanisms in *F. graminearum* with laboratory-induced mutants because this helps to determine the potential risks in the field associated with fungicide resistance in the future.

Among the fungicides, phenylpyrroles also play an important role in controlling *F. graminearum* in the field. Although this has been identified as an efficient fungicide, several studies have been reported about phenylpyrrole-resistant *F. gramineraum* isolates and their resistance mechanisms [40,42]. The study conducted by Wen et al. [40] identified several numbers of fludioxonil-resistant isolates collected from fields in three different counties in China and found 0.3%, 1.42%, and 6.64% frequencies of fludioxonil-resistant isolates from Jiangsu, Anhui, and Henan counties, respectively. Although the sequence analysis of the target gene identified several mutations in the fludioxonil-resistant isolates, it is not yet clear whether these mutations are directly associated with fludioxonil resistance. This was further proved by the study conducted by Shi et al. [42], where the comparison of the whole genome sequences between the fludioxonil mutants and parents had no mutations associated with fludioxonil resistance. But, interestingly, it was found that the overexpression of the tyrosine-protein phosphatase gene, *FgPtp3,* in the MAPK pathway is associated with fludioxonil resistance. Although the resistance screenings were performed by targeting only fludioxonil resistance, it is also important to check the possibility of having multiple resistances in *F. graminearum* isolates between fludioxonil and other fungicides. To investigate this, Wen et al. [41] created laboratory-induced mutants of *F. gramienearum* having dual resistance to both fludioxonil and phenamacril fungicides and found that these dual-resistant isolates are genetically stable over many generations. However, the lower fitness in the phenotypes of the dual-resistant isolates indicated that there is still a low risk of developing dual resistance in field isolates.

## 5. Mitigation Strategies for the Development of Fungicide Resistance in the Pathogen Population

Although fungicides can effectively control FHB in the field, fungicide insensitivity or resistance is also being frequently reported. Thus, it is important to find the reasons for fungicide resistance and find the strategies to delay fungicide resistance developing in the pathogen population while using them at the correct level that is enough to control the disease in the field effectively.

Monitoring the pathogen population for fungicide sensitivity is important to perform yearly. Monitoring the pathogen population for resistance can be performed by collecting representative samples from wheat fields and screening the pathogen population for different fungicides [76,104,105]. Monitoring fungicide sensitivity gives an early warning about the fungicide sensitivity shift in the pathogen population towards resistance. However, this monitoring will not be fully applicable for monitoring single-step resistance where the resistance happens directly from sensitive to resistant in one step. But, this still works for single-step resistance when the sample size is enough to detect 1% resistant frequency in the pathogen population [63]. However, with multi-step resistance, insensitive pathogen strains can be commonly found in the population. Therefore, insensitive pathogen strains can be easily detected through fungicide monitoring systems, which helps identify the risk of fungicide resistance ahead [63]. Frequent monitoring of the pathogen population for fungicide sensitivity over the years will be helpful in the future in developing fungicide monitoring programs for the *F. graminearum*–FHB pathosystem [76]. Monitoring the pathogen population for fungicide sensitivity is important not only to set up an early warning but also to confirm that the disease is efficiently controlled by chemical control [63].

The main factor for developing fungicide resistance in the pathogen population is the extensive use of fungicides in the field over many years. Therefore, certain precautions should be taken to avoid overuse of fungicides. As an attempt to reduce the overuse of fungicides in the field, it is recommended to follow the manufacturing recommendations, use a minimum number of sprays within the growing season, use a combination/mixture of fungicides having different modes of action, and follow disease forecasting models [63,106]. Several countries have developed these disease forecasting systems for FHB and contribute to predicting the full spike emergence, DON accumulation, and other risk thresholds by considering phenological, epidemiological, and weather data [106,107]. Although several forecasting models are available, several factors such as climate, year, and location effects can be limiting factors for not being able to use the same models everywhere [108,109]. However, the optimized disease forecasting systems help the growers to decide the necessity of fungicide applications, frequency, and fungicide timing accurately, thus preventing the overuse of fungicides [106]. All these precautions may help to decrease the selection pressure in the pathogen population towards fungicide resistance, thus delaying the fungicide resistance process throughout the years in the field. In addition, integrated pest management (IPM) is also important not only in controlling FHB but also in delaying fungicide resistance. It is also important to identify the risk of overusing fungicides in developing fungicide resistance as well as food security. The Green Deal in the European Union is one of the examples aiming to reduce the risk of pesticides, including fungicides, by helping farmers use the pesticides properly and follow integrating pest management [https://ec.europa.eu/stories/european-green-deal/, accessed on 7 November 2024]. The IPM can be achieved by combining all the factors that control FHB pressure, such as the use of resistant cultivars, cultural practices, biological control agents, and maintaining the disease under control. Presently, several potential biological controls with *Baccillus* spp. *Streptomyces* spp. *Pseudomonas* spp., *Cryptococcus* spp., and *Clonostachys* spp. have been identified that can effectively control *F. graminearum* [110,111,112,113,114]. Among them, some are available in the market as bio-fungicides [115]. Although the efficacy of the biocontrols is still questionable, the study conducted by Xue et al. [110] found no statistical difference between the bio-fungicide CLO-1 and the conventional fungicides on FHB index, DON content, and the Fusarium damaged kernels (FDKs). Although biological control alone is not as effective as the conventional fungicides, it is important to integrate biocontrol along with chemical control to keep the fungicide application dose at a minimum. Altogether, the integrated management strategies along with fungicides are important to decrease the frequency of using the fungicide and lead to the delay of the fungicide resistance in the pathogen population by slowing down the selection pressure for the fungicides [63]. Moreover, several studies have tested the effect of essential oil on *F. graminearum* control [116,117,118,119,120]. Essential oils are plant-based chemicals that include mono, di, and sesqui terpenes and other derivatives, and they possess high antifungal properties [120]. It has been reported that the essential oil extracted from different crops, including thymus, oregano, and basil, showed antifungal activity against *F. graminearum* by inhibiting mycelium growth [120]. In addition, essential oils such as orange oil reduced the mycotoxin accumulation in wheat grains by FHB pathogens [118]. Therefore, essential oils are also considered potential antifungal agents to apply against *F. graminearum*. In addition, finding alternatives for fungicides is also necessary. Currently, with the massive development of nanotechnology, it has been found that engineered nanoparticles have the capability of acting as antimicrobial agents [121]. Currently, several types of nanoparticles such as silver, zinc oxide, silica, and chitosan have been identified to effectively control *F. graminearum* isolates [121,122,123,124,125]. For instance, the study conducted by Kheiri et al. [125] found that the application of the chitosan nanoparticles reduced the mycelial growth and spore germination of *F. graminearum* isolates *invitro,* and the greenhouse inoculation trials showed the chitosan nanoparticle-treated plants had low disease severity when chitosan was applied before the pathogen inoculation. Furthermore, the study conducted by Jian et al. [121] tested the efficacy of silver nanoparticles against fungicide-resistant *F. graminearum* isolates and found that they effectively control both azole-resistant and sensitive *F. graminearum* isolates. Therefore, the use of nanoparticles will be able not only to integrate with fungicide application to control FHB pressure, but also to control fungicide-resistant isolates in the field, so it helps to enhance the longevity of the fungicide.

Since exposure to the same fungicide helps accelerate the development of fungicide resistance in the pathogen populations, alternative strategies should be followed to prevent constant exposure to the same fungicide over the years. In disease management, crop rotation is one of the effective methods of controlling disease pressure in the field and helps reduce the primary inoculum in the field. The same scenario can be applied in fungicide management by rotating the fungicides with different action modes [63,126]. Although fungicide rotation has not been reported on the FHB pathosystem, other pathosystems, such as watermelons against the Phytophthora root rot, have proven that certain fungicide rotations have been effectively controlling the disease [126]. Therefore, it will be helpful to develop fungicide rotation programs for the FHB pathosystem.

## 6. Conclusions and Future Remarks

FHB management through chemical control plays a significant role among the control strategies recommended to control FHB. Although host resistance plays a vital role in FHB management, a strong host resistance was not commonly found in some varieties, including durum wheat. Thus, chemical control is crucial to control FHB with a lack of host resistance. Several fungicides have been registered to control FHB and the disease in the field. However, fungicide resistance is one of the problems that arises with the overuse of the same fungicides. Therefore, precautions should be taken to delay the fungicide resistance development in the pathogen and strictly follow the manufacturer’s recommended dose, restrict the overuse of the fungicide by reducing the frequency of fungicide application, and change the fungicide classes over years through fungicide rotation programs, and development of integrated management strategies are needed to delay the fungicide resistance in the pathogen population.

Although DMIs have been identified as an effective fungicide to control *F. graminearum*, there is a risk of developing fungicide resistance because they have been used consistently over the years in several countries, such as Canada. Although many studies have reported that DMI resistance is rare, monitoring the pathogen population for fungicide sensitivity is always better. Therefore, establishing fungicide sensitivity monitoring programs for *F. graminearum* is needed to identify the fungicide-resistant risk in the future.

The fungicide rotation concept will help reduce the selection pressure in the pathogen population toward fungicide resistance to delay the development of fungicide resistance in the pathogen population. However, no studies have been conducted on the effect of fungicide rotations on *F. graminearum*. Therefore, more studies should be conducted on fungicide rotation on the FHB pathosystem, and this will help in the future not only to delay the resistance towards already effective fungicides but also to reuse the ineffective fungicides when the selection pressure is broken down towards that fungicide.

It has been reported that several fungicide resistance mechanisms have been established in *F. graminearum* against certain fungicides. Although the current approaches to detecting fungicide resistance mechanisms such as mutation and overexpression analysis are available, they are generally time-consuming, Regarding the *F. graminearum*-FHB pathosystem, a rapid detection method with a mismatch allele-specific polymerase chain reaction (MAS-PCR) was developed to identify the point mutation in the succinate dehydrogenase inhibitor for fungicide-resistant *F. graminearum* isolates. Identifying fungicide resistance isolates through a rapid detection method is helpful for growers and decision-makers during monitoring. Therefore, more studies should be conducted on developing rapid diagnostic techniques to identify resistant *F. graminearum* isolates for other common fungicides, such as benzimidazoles, DMIs, and QoIs.

## Figures and Tables

**Table 1:** Some common fungicides for FHB control and their mode of action and target sites.

Group	Chemical/Biological Name	Mode of Action	Target Site	Frac Code	References
DMI (Demethylase inhibitors)	Triazoles, Imidazoles	Sterol biosynthesis in plasma membrane	*Cyp51/erg11* C14 demethylase in sterol biosynthesis	3	[24,27]
Qoi fungicides (Quinone outside inhibitors)	Methoxy-acrylates	Respiration	Cytochrome c Respiration	11	[24,27,28,29,30,31,32]
MBC fungicides	Benzimidazoles	Cytoskeleton and motor protein	Tubulin polymerization	1	[33,34,35,36]
Cyanoacryates	aminocyanoacryates	Cytoskeleton and motor protein	Actin/myosin/fimbrin function	47	[37]
SDHI fungicides (Succinate dehydrogenase inhibitors)	N-methoxy-(phenyl-ethyl)-pyrazole-carboxamides	Respiration	Complex II: succinate dehydrogenase	7	[38,39]
PP-fungicides (phenylpyrroles)	phenylpyrroles	Signal transduction	MAP/Histidine-kinase in osmotic signal transduction	12	[40,41,42]
